# Tuning the electronic transport properties of grapheme through functionalisation with fluorine

**DOI:** 10.1186/1556-276X-6-526

**Published:** 2011-09-12

**Authors:** Freddie Withers, Saverio Russo, Marc Dubois, Monica F Craciun

**Affiliations:** 1 Centre for Graphene Science, College of Engineering, Mathematics and Physical Sciences, University of Exeter, Physics building, Exeter EX4 4QF, UK; 2 Clermont Université, UBP, Laboratoire des Matériaux Inorganiques, CNRS-UMR 6002, 63177 Aubière, France; 3 Centre for Graphene Science, College of Engineering, Mathematics and Physical Sciences, University of Exeter, Harrison building, Exeter EX4 4QL, UK

## Abstract

We demonstrate the possibility to tune the electronic transport properties of graphene mono-layers and multi-layers by functionalisation with fluorine. For mono-layer samples, with increasing the fluorine content, we observe a transition from electronic transport through Mott variable range hopping (VRH) in two dimensions to Efros-Shklovskii VRH. Multi-layer fluorinated graphene with high concentration of fluorine show two-dimensional Mott VRH transport, whereas CF_0.28 _multi-layer flakes exhibit thermally activated transport through near neighbour hopping. Our experimental findings demonstrate that the ability to control the degree of functionalisation of graphene is instrumental to engineer different electronic properties in graphene materials.

## 1 Introduction

**Figure 1:**
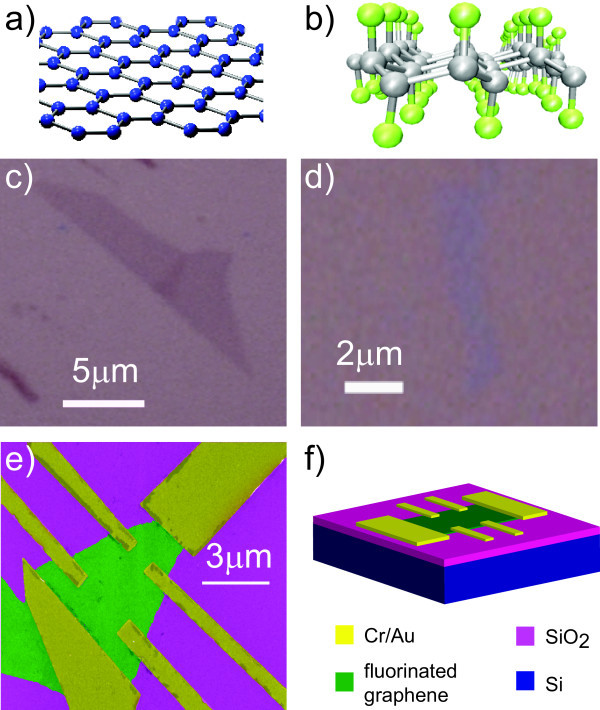
**Fabrication of fluorinated graphene layers and transistor devices**. Crystal structure of pristine graphene **(a) **and fluorinated graphene **(b)**. The grey balls in **(b) **represent the carbon atoms, whereas the green balls are the fluorine atoms. Optical image of pristine graphene **(c) **and of fluorinated graphene **(d)**. **(e) **False colour SEM image of a fluorinated graphene device. **(f) **Schematic view of the transistor structure fabricated on fluorinated graphene.

Graphene, a mono-layer of *sp*2 bonded carbon atoms arranged in a honeycomb pattern (Figure 1a), is a two-dimensional semi-metal where the valence and conduction bands touch in two independent points at the border of the Brillouin zone, named K and K' valleys [1-5]. This material has remarkable electronic, optical and mechanical properties which can be used in a new generation of devices [6,7]. For instance, the high mobility of charge carriers is attracting considerable interest in the realm of high-speed electronics [8]. Furthermore, thanks to the unique combination of high electrical conductivity [4,5] and optical transparency [9], graphene is a promising material for optoelectronic applications such as displays, photovoltaic cells and light-emitting diodes. Few-layer graphene are yet unique materials [10] with unprecedented physical properties: bilayers are semiconductors with a gate-tuneable band gap [11-21], whereas trilayers are semi-metals with a gate-tuneable overlap between the conduction and valence bands [22,23]. However, the use of graphene for applications in daily-life electronics suffers from a major drawback, i.e. the current in graphene cannot be simply pinched off by means of a gate voltage. A valuable solution to this problem is to engineer a band gap in the energy spectrum of graphene for example confining the physical dimensions of graphene into nanoribbons [24-28] or by chemical functionalisation [29-46].

When chemical elements, e.g. oxygen, hydrogen or fluorine, are adsorbed on the surface of graphene, they form covalent bonds with the carbon atoms. As a result, the planar crystal structure of graphene characterised by *sp*2 bonds between the carbon atoms is transformed into a three-dimensional structure with *sp*3 bonds (see Figure 1b). The adsorbed elements can attach to graphene in a random way, as it is the case in graphene oxide [44-46], or they can form ordered patterns as it has been found for hydrogen [33-35] and fluorine [36-43] adsorbates. *Ab initio *calculations performed within the density functional theory formalism predict that functionalisation with hydrogen and fluorine should lead, respectively, to a band gap of 3.8 and 4.2 eV for full functionalisation [29-32].

Successful hydrogenation and fluorination of graphene have been recently achieved by several groups [33-43]. Hydrogenation is usually carried out in a remote plasma of H_2 _[33-35] which makes it difficult to control the degree of induced atomic defects as well as the stoichiometry of the functionalisation. Furthermore, hydrogenated graphene can loose H at moderate temperatures [33], which limits the use of this material in applications where high-temperature stability is required. On the other hand, fluorine has higher binding energy to carbon and higher desorption energy than hydrogen [29-32]. Opposed to hydrogenation, the process of fluorination is easy to control, e.g. *via *temperature and reactant gases, leading to reproducibly precise C/F stoichiometries.

Here, we explore the electronic transport properties of functionalised graphene with a fluorine content ranging from 7% (i.e. CF_0.07 _or F/C atomic ratio of 0.07) to 100% (CF_1_). We have fabricated transistor structures with fluorinated graphene mono-layers and multi-layers and studied their electrical transport properties in the temperature range from 4.2 to 300 K. We show that the electronic transport properties of fluorinated graphene can be tuned by adjusting the fluorine content, so that different transport regimes can be accessed, like Mott variable range hopping (VRH) in two dimensions [47,48], Efros-Shklovskii VRH [49] and nearest neighbour hopping (NNH) transport.

## 2 Experimental details

Fluorinated graphene mono-layers and multi-layers were mechanically exfoliated from fluorinated graphite and deposited onto conventional Si/SiO_2 _(275 nm) substrates. The fluorinated graphite was synthesised via two routes: graphite fluorides (using F_2 _gas) and fluorine graphite intercalation compounds (FGIC) (using XeF_2 _as fluorinating agent), see Section 6. The samples produced using F_2 _gas that we investigate here are multi-layers and have the concentration of fluorine of 28 and 100%, whereas the samples synthesised using XeF_2 _gas are all mono-layers and have the fluorine content of 7, 24 and 28%.

**Figure 2:**
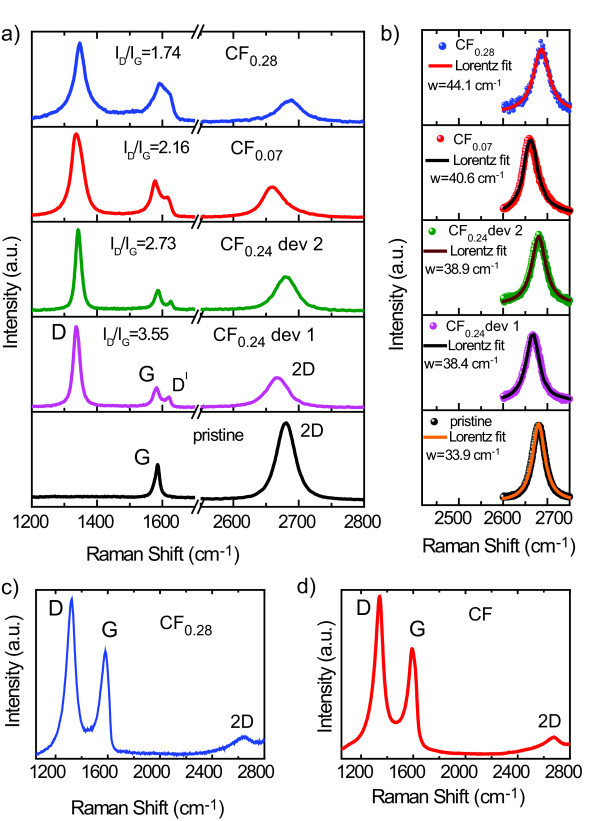
**Raman spectroscopy in fluorinated and pristine graphene**. **(a) **Raman spectra of mono-layer fluorinated graphene with different fluorine content and pristine mono-layer graphene. **(b) **Fitting of the 2D peak with a single Lorentzian function for pristine and fluorinated mono-layer graphene. **(c, d) **Raman spectra of fluorinated multi-layer graphene.

Flakes of fluorinated graphene are located using an optical microscope (see Figure 1d) and subsequently characterised by Raman spectroscopy. Mono-layer graphene flakes were identified by fitting the 2D peak of the Raman spectra by a single Lorentzian function (see Figure 2b), with a full width at half maximum (FWHM) of 30-45 cm^-1 ^which is typical for pristine mono-layer graphene [50]. The height of the studied multi-layer flakes is determined by Atomic Force Microscopy: 10-nm height for flakes exfoliated from the CF and 0.86-6.1 nm for flakes obtained from CF_0.28_. In total, four mono-layer, five CF and five CF_0:28 _multi-layer flakes were processed into four-terminal transistor devices, where the electrical contacts were defined by e-beam lithography, deposition of Cr/Au (5/50 nm) and lift-off procedure, see Figure 1e,f.

The typical optical contrast of fluorinated graphene is ~2-6%, which is systematically lower than what we observe on pristine graphene (~9%), see Figure 1c,d. The reduced contrast in fluorinated graphene has to be expected, since the opening of a large energygap in the energy dispersion of fluorinated graphene lowers the optical absorption transitions between conduction and valence bands.

## 3 Raman spectroscopy

Figure 2 shows the Raman spectra of a mechanically exfoliated pristine graphene flake, with the G and 2D (also known as G') bands at 1580 and 2700 cm^-1^. The G band is associated with the double degenerate E2g phonon mode at the Brillouin zone center, while the 2D mode originates from a second-order process, involving two intervalley phonons near the K point, without the presence of any kind of disorder or defect [50]. In the fluorinated graphene, additional peaks are activated in the Raman spectra (see Figure 2), the D and D' peaks that appear at 1350 and 1620 cm^-1^. These Raman peaks originate from double-resonance processes at the K point in the presence of defects, involving, respectively, intervalley (D) and intravalley (D') phonons [51-54].

In exfoliated pristine graphene, the D peak can only be observed at the edges of the flakes where there is a large concentration of structural defects and its intensity is typically much lower than the intensity of the G peak [55,56]. In our studies performed on pristine graphene flakes with similar size as the fluorinated graphene flakes, the intensity of the D peak is typically well below the sensitivity of our Raman setup, i.e. we are usually not able to detect any D peak because of the edges of the flakes. Therefore, the observed D peak in our fluorinated graphene samples must originate from other defects than simply the edges of the samples. As all our samples contain networks of *sp*2 bonded carbon atom rings, we believe that the D peak is mainly activated by the F atoms which act as vacancies in these *sp*2 rings.

A better understanding of the level of disorder in our samples is reached when analysing the intensity ratio I_D_/I_G _for the D and G bands. It has recently been shown that in graphene I_D_/I_G _has a non-monotonic dependence on the average distance between defects L_D_, increasing with increasing L_D _up to L_D _~ 4 nm and decreasing for L_D _> 4 nm [53,54,57]. Such behavior has been explained by the existence of two disorder-induced regions contributing to the D peak: a structurally disordered region of a radius ~1 nm around the defect and a larger defect-activated region which extends to ~3 nm around the defect. In the defect-activated region, the lattice structure is preserved, but the proximity to a defect causes a mixing of Bloch states near the K and K' valleys. Consequently, the breaking of the selection rules leads to an enhancement of the D peak. Furthermore, it was shown that in the structurally disordered region, the G and D' peaks overlap.

The Raman spectra of fluorinated mono-layer samples produced from graphite with fluorine content of 7 and 28% (see Figure 2a) systematically show that the G and D' peaks have a significant overlap. On the other hand, the samples exfoliated from CF_0.24 _exhibit very distinct G and D' peaks. Based on the aforementioned phenomenological model [53,54,57], we can state that the CF_0.07 _and CF_0.28 _samples are in the regime where the intensity ratio I_D_/I_G _increases with increasing L_D _(i.e. decreasing the concentration of F) whereas the CF_0.24 _samples are in the opposite regime. This scenario is confirmed when comparing the intensity ratio I_D_/I_G _for fluorinated mono-layers extracted from graphite with different fluorine content: I_D_/I_G _= 1.74 for CF_0:28 _and I_D_/I_G _= 2.16 for CF_0.07_. From the L_D _dependence on I_D_/I_G _we estimate L_D _~ 1.5 nm for CF_0.28 _and L_D _~2 nm for CF_0.07 _[53,54,57]. For the CF_0.24 _samples, we estimate L_D _~ 5.3 nm for device 1 and L_D _~ 6.1 nm for device 2. These values of L_D _are in agreement with the observed frequencies of the D, G, D' and 2D peaks as well as with the FWHM values of the 2D peaks [53].

In the case of the fluorinated multi-layer flakes, see Figure 2c,d, it is difficult to perform a similar analysis, as the intensity of the G band depends on the number of graphene layers present in the sample [50]. For samples thicker than three to four layers, the structure of the 2D peak does not provide an accurate estimation for the number of layers because of the large number of fitting parameters.

## 4 Electrical transport measurements

**Figure 3:**
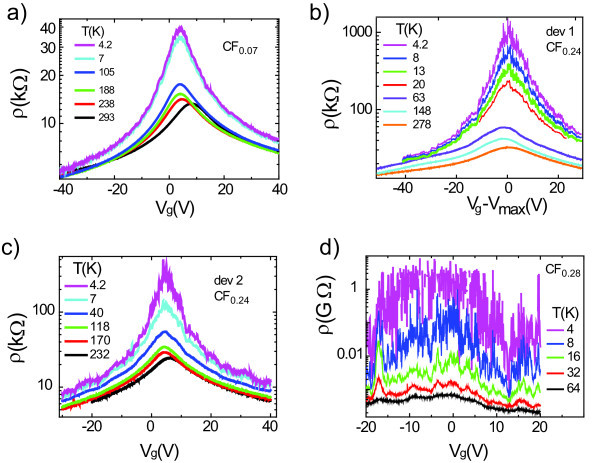
**Electronic transport in fluorinated mono-layer graphene**. Resitivity of four fluorinated mono-layer graphene samples as a function of gate voltage at different temperatures. The different panels correspond to different concentration of fluorine in the starting fluorinated graphite material, as indicated in each panel.

Having characterised the level of disorder from Raman spectroscopy, we now proceed to address the role of disorder on the electrical transport properties of fluorinated graphene materials. Figure 3 shows the resistivity (*ρ*) as a function of gate voltage (*V*_g_) for the fluorinated mono-layer samples for different temperatures. The resistivity exhibits a non-monotonous dependence on *V*_g _with a maximum at *V*_g _= +10 V, stemming for a doping level of *n *= 0.74 10^12 ^cm^-2 ^commonly seen also in pristine graphene devices and attributed to doping by atmospheric water. In all cases, the resistivity of fluorinated graphene shows a pronounced temperature dependence. Indeed, the maximum of resistivity changes over two orders of magnitude as *T *decreases from 300 to 4.2 K. Away from the maximum of resistivity region, the temperature dependence remains weak, with the mobility of carriers of 150 cm^2^/Vs. Furthermore, at low temperature the resistance shows strong mesoscopic fluctuations, as expected for samples of small size [58]. In the analysis of the maximum of resistivity, we smooth the *ρ*(*V*_g_) curves using a moving average filter.

**Figure 4:**
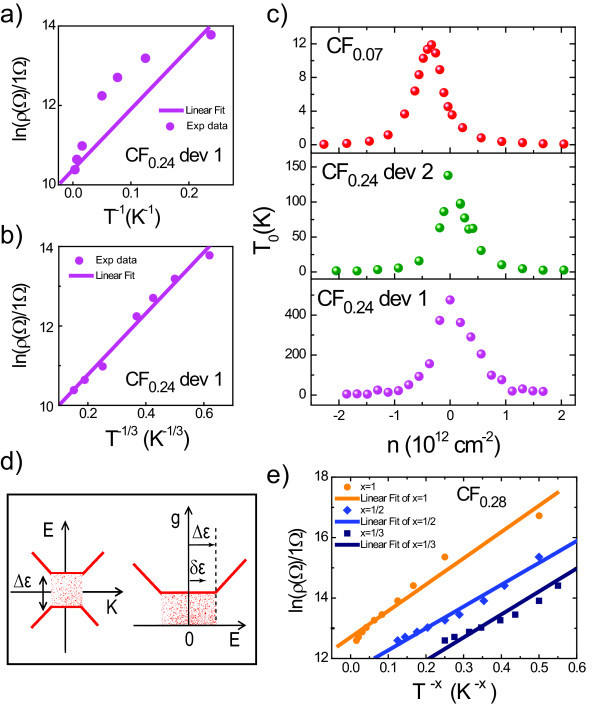
**VRH transport in fluorinated mono-layer graphene**. Resistivity of fluorinated mono-layer graphene in the charge neutrality region plotted as a function of *T*^-1 ^**(a) **and T^-1/3 ^**(b)**. **(c) **The values of the hopping parameter *T*_0 _as a function of carrier density for the samples where transport occurs by two-dimensional Mott VRH. **(d) **Schematic diagrams of the energy dispersion of fluorinated mono-layer graphene (left panel) and of the energy dependence of the density of electron states, with the Fermi level at zero energy (right panel). The localised states are shown by the shaded area. **(e) **Resistivity of fluorinated mono-layer graphene (exfoliated from CF_0.28 _graphite) in the charge neutrality region plotted as a function of T^-*x*^. The solid lines represent fits to the experimental data where *x *= 1 for thermally activated transport, *x *= 1/2 for Efros-Shklovskii VRH and *x *= 1/3 for 2D Mott VRH. The best t is obtained for *x *= 1/2.

To examine the presence of the energy gap, we analyse the temperature dependence of the maximum of resistivity by an exponential law describing thermal activation of carriers across an energy gap *Δε*: *ρ*(*T*) = *ρ*_0_*exp*(*Δε/*2*k*_B_T), see Figure 4a. This analysis clearly shows that our data are not described by the thermal activation law over the whole temperature range. We note that the slope of *lnρ *(1/*T*) versus 1/*T *decreases with decreasing *T*, which is a signature of hopping conduction via localised states [48]. The fact that in the whole range of studied temperatures electron transport is not due to thermal activation across the gap but due to hopping becomes clear when re-analysing the temperature dependence in terms of the 2D Mott VRH (2D-VRH) [47,48]. In this model, the functional dependence of *ρ *on temperature is *ρ*(*T*) = *ρ*_0_*exp*(*T*_0_/*T*)^1/3^, where *k*_B_*T*_0 _= 13.6/*a*^2^*g*(*ε_F_*), *g *is the density of localised states at the Fermi level *ε_F _*and *a *is the localisation length [47,48]. Experimentally we find that the measured *ρ*(*T*) for the samples produced from CF_0.07 _and CF_0.24 _graphite (see Figure 3b for CF_0.24_) is described well by the 2D-VRH model.

Figure 4c shows the hopping parameter *T*_0 _as a function of carrier concentration for these three samples. The value of *T*_0 _approaches zero at a carrier concentration of ±1.2 10^12 ^cm^-2^. This value gives the concentration of the localised electron states in the energy range from *ε *= 0 to the mobility edge, see Figure 4d. The mobility edge occurs at *V*_g _± 20*V *and indicates the transition from hopping to metallic conduction.

In order to relate the obtained concentration of the localised states to the energy gap *Δε*, one needs to know the exact energy dependence of the density of states in the gap. For estimations, we will use the linear relation for the density of extended states above the mobility edge *g*(*ε*) = 2*ε*/*πħ*^2^*v*^2 ^(*v *= 10^6 ^m/s is the Fermi velocity), and a constant value for the density of localised states below the mobility edge, Figure 4d. This gives *Δε *= 60 meV and twice this value for the full mobility gap. In this approximation, the density of the localised states in the gap is ~10^36 ^J/m^2^. Using the obtained value of the hopping parameter at the maximum of resistivity, we can then estimate the localisation length at *ε *= 0 as *a *= 40 nm for CF_0.24 _(device 1), *a *= 81 nm for CF_0.24 _(device 2) and *a *= 265 nm for CF_0.07_.

Figure 4e shows the analysis of the temperature dependence of the resistivity for fluorinated mono-layer graphene exfoliated from CF_0.28 _graphite. For this sample, characterised by the largest disorder L_D _~1.5 nm, the experimental data cannot be described by thermally activated law nor Mott VRH. In this case, the *ln*(*ρ*) follows a *T*^-1/2 ^dependence characteristic of the Efros-Shklovskii VRH in the presence of Coulomb interaction between the localised states (*ρ*(*T*) = *ρ*_0_*exp*(*T*_0_/*T*)^1/2^) [49]. *T*_0 _is related to the localisation lengths through *T*_0 _= 2.8e^2^ / 4 *πε_r_ε*_0_*k*_B_*a *and for our sample we estimate *T*_0 _= 52 K. Assuming that *ε_r _*is the dielectric constant of SiO_2 _we obtain the localisation length *a *= 282 nm.

**Figure 5:**
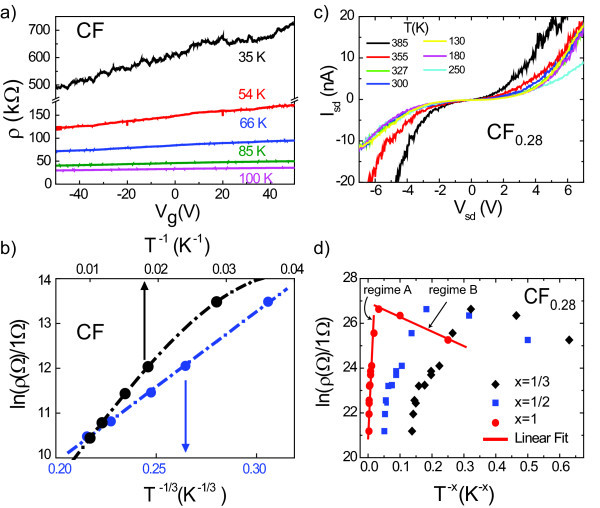
**Electronic transport in fluorinated multi-layer graphene**. **(a) **Resistivity as a function of gate voltage for an annealed fully fluorinated device. **(a) **Resistivity for the fully fluorinated device plotted against *T*^-1 ^and *T*^-1/3 ^at *V*_g _= +50 V. **(c) ***I*-*V *characteristics for multi-layer fluorinated graphene exfoliated from CF_0.28 _graphite. **(d) **Resistivity of fluorinated multi-layer graphene (exfoliated from CF_0.28_) plotted as a function of *T*^-*x*^, where *x *= 1 for thermally activated transport, *x *= 1/2 for Efros-Shklovskii VRH and *x *= 1/3 for 2D Mott VRH.

We turn now our discussion to multi-layer fluorinated graphene exfoliated from fully fluorinated graphite and from CF_0.28 _prepared by exposure to fluorine gas. The fully fluorinated multi-layer show systematically a very large resistance (more than 100 GΩ) independently of the specific thickness and no gate-voltage control of the resistivity. To achieve gate modulation in these samples, we reduced the fluorine content by annealing the samples at 300°C, in a 10 % atmosphere of H_2_/Ar for 2 h. This annealing process restores a partial gate-voltage control of the resistance (Figure 5a) while leaving unchanged the Raman spectrum in Figure 2d.

Resistance measurements of CF*_n _*flakes after annealing show a strong temperature dependence, Figure 5a. Analysis of the temperature dependence of the resistance in terms of the activation law at the highest gate voltage *V*_g _= 50 V (which is still far from the Dirac point) gives a gap of only 25 meV, which is significantly smaller than the expected energy gap for fully fluorinated graphene. Similarly to the fluorinated mono-layer graphene, the resistivity dependence on temperature is fitted well by VRH with the value of *T*_0 _= 20000 K. This confirms that the previously found activation energy of 25 meV is not the activation energy *Δε *that separates the localised states from extended states at the mobility edge, but is an activation energy δ*ε *of hopping between localised states within the mobility gap, see Figure 4d.

Figure 5c,d shows the transport data for the multi-layer fluorinated graphene CF_0.28 _prepared by exposure to fluorine gas. The *I*-*V *characteristics of these samples are strongly non-linear (see Figure 5c) with resistances of more than 1 GΩ. In this case, the dependence of the resistivity on temperature cannot be described neither by 2D-VRH nor by Efros-Shklovskii VRH (see Figure 5d). The ln(*ρ*)(*T*) dependence is rather well described by a thermally activated law at elevated temperatures (regime A), with a *Δε *= 0.25 eV, followed by a temperature regime where the resistivity decreases with lowering the temperature (regime B). A 1/*T *dependence of ln(*ρ*) could be the consequence of either intrinsic transport through thermally excited carriers above the bandgap or conduction through NNH via localised states within the gap. Since the *I*-*V *characteristics of our devices show a non-linearity on a *V*_sd _range much larger than the estimated *Δε *= 0.25 eV (see Figure 5c), we conclude that transport occurs via NNH.

Finally, for the same degree of fluorination (i.e. CF_0.28_) the graphene multi-layers exhibit a stronger temperature dependence and a larger transport gap than what is observed in mono-layers. This difference could originate from the different fluorination processes used for the multi-layers (direct fluorination with F_2 _gas at high temperature to produce graphite fluorides) and for the monolayers (FGIC synthesised at lower temperatures), see Section 6. Indeed, different fluorination processes may lead to different concentrations of localised states. Even though in both materials, F-GIC and graphite fluorides, the nature of the bonding between fluorine and carbon atoms is covalent, the C-F bond order is slightly lower in F-GIC [59]. The lower C-F bond order in F-GIC is due to the hyper-conjugation which occurs between the C-F and the C-C single bonds around the C-F bonds. In particular, the C-F and C-C single bond lengths are, respectively, longer and shorter in F-GIC than those in graphite fluorides. As a result, the electrons involved in the covalent C-F bonds in F-GIC are slightly delocalised by this hyperconjugation, which may result in a smaller transport gap.

## 5 Conclusions

In conclusion, we have demonstrated the possibility to tune the band structure and therefore the electronic transport properties of graphene through functionalisation with fluorine. In particular, depending on the fluorine concentration different transport regimes can be accessed. For mono-layer samples, we observe a transition from 2D Mott VRH to Efros-Shklovskii VRH with increasing the fluorine content. Multi-layer fluorinated graphene with high concentration of fluorine shows 2D Mott VRH, whereas CF_0:28 _multi-layer flakes exhibit NNH transport. Our experimental findings demonstrate that the ability to control the degree of functionalisation of graphene is instrumental to engineer different electronic properties in graphene materials. In all cases, fluorinated graphene transistors exhibit a large on/off ratio of the current, making this material of interest for future applications in transparent and bendable electronics.

## 6 Methods

### 6.1 Fluorination of graphite

To produce fluorinated graphite, we have used two distinct methods [59-62]. In the first method, graphite is heated in the presence of F_2 _to temperatures in excess of 300°C, so that covalent C-F bonds are formed and modify the carbon hybridisation [60]. The layered structure of graphite is then transformed into a 3D arrangement of carbon atoms (Figure 1a,b). In this article, we present the studies on graphene exfoliated from fully fluorinated HOPG graphite CF*_n _*(obtained at 600°C) and CF_0.28 _synthesised with this method at 530°C. However, due to the harsh fluorination conditions, many structural defects are formed, which makes it very difficult to exfoliate large enough mono-layer flakes that can be identified by optical microscopy and easily processed into devices. To prepare larger fluorinated graphene samples, we have used a second fluorination method where graphite is exposed to a fluorinating agent, i.e. XeF_2_. In this case, the functionalisation process is carried out at *T *≤ 120°C, as XeF_2 _easily decomposes on the graphite surface into atomic fluorine [59]. The mixture of natural graphite and XeF_2 _was prepared in a glove box in an Ar atmosphere. Owing to its reactivity and diffusion, the fluorination results in a homogenous dispersion of fluorine atoms that become covalently bonded to carbon atoms [59,62,63]. At low fluorine content, the F/C atomic ratio is ≤ 0.4. In this case, the conjugated C-C double bonds in the non-fluorinated parts and covalent C-F bonds in corrugated fluorocarbon regions coexist [62,64]. The concentration of the covalent bonds increases with increasing the concentration of fluorine. The samples produced using the XeF_2 _gas that we investigate here have the concentration of fluorine of 7, 24 and 28%.

### 6.2 Determination of fluorine concentration

The fluorine concentration (i.e. F:C molar ratio) of fluorinated graphite was determined by gravimetry (weight uptake). The concentration obtained by weight uptake was confirmed by solid-state NMR measurements on samples fabricated under identical conditions and the accuracy of gravimetry was estimated to be 0.02 [65-67]. The fluorine concentration measured by gravimetry can be under-estimated due to the decomposition of graphite under fluorine gas at high temperature, which results in the formation of carbene (CF_2_) and C_2_F_4_. However, the decomposition of graphite was found to start as a secondary reaction close to 600°C, with fluorination being the main reaction. Since the reactions with F_2 _and XeF_2 _have been carried at lower temperatures than the graphite decomposition temperature, the underestimation of F:C ratio in our fluorinated graphite samples is likely to be less than 0.02.

### 6.3 Raman spectroscopy characterisation

We have characterised all the exfoliated flakes by Raman spectroscopy using an excitation light with a wavelength of 532 nm and a spot size of 1.5 *μ*m in diameter. An incident power of 5 mW was used. We ensured that this power does not damage the graphene by performing Raman measurements on a similarly sized pristine graphene flake which shows the common spectra of mechanically exfoliated graphene: the G band and 2D band (also known as G') at 1580 and 2700 cm^-1^, see Figure 2.

### 6.4 Electrical characterisation

The resistance of the transistor devices was measured both in dc, by means of Keithley 2400 Source-meter, and in ac at low frequency (34 Hz) with a lock-in amplifier in a voltage-biased configuration. For the ac-measurements, the excitation current was varied to ensure that the resulting voltage was smaller than the temperature to prevent heating of the electrons and the occurrence of nonequilibrium effects. The comparison of 2- and 4-probe measurements shows that the contact resistance in our devices is negligible as compared to the sample resistance. This experimental finding insures that even 2-probe transport measurements are probing the electrical properties of the bulk fluorinated graphene rather then simply the Cr/graphene interface.

## Abbreviations

2D-VRH: two-dimensional Mott variable range hopping; AFM: atomic force microscopy; F-GIC: fluorine graphite intercalation compounds; FWHM: full width at half maximum; NMR: nuclear magnetic resonance; NNH: nearest neighbour hopping; VRH: variable range hopping.

## Competing interests

The authors declare that they have no competing interests.

## Authors' contributions

FW fabricated the fluorinated graphene devices and carried out the measurements. MD synthesised the fluorinated graphite. FW and MFC performed the data analysis. FW, SR and MFC wrote the manuscript. All authors discussed the results, read and approved the final manuscript.
